# Consumer perspectives on the national electronic health record and barriers to its adoption in Germany: does health policy require a change in communication?

**DOI:** 10.1186/s12913-024-12175-6

**Published:** 2025-01-07

**Authors:** Saskia Kröner, Björn Schreiweis, Veronika Strotbaum, Lea Christine Brandl, Monika Pobiruchin, Martin Wiesner

**Affiliations:** 1 Health Informatics Research Group, University AS Osnabrück, Osnabrück, Germany; 2 Institute for Medical Informatics and Statistics, University Hospital Schleswig-Holstein and Kiel University, Kiel, Germany; 3 Consumer Health Informatics special interest group of the German Association for Medical Informatics, Biometry and Epidemiology (GMDS e. V.), Cologne, Germany; 4 Institute of Telematics, University of Lübeck, Lübeck, Germany; 5 Heilbronn University, Heilbronn, Germany

**Keywords:** Adoption, Barriers, National electronic health record, Communication, Consumers

## Abstract

**Background:**

The national health record (ePA) was introduced January 1^st^, 2021 in Germany and is available to every person insured under statutory health insurance. This study investigated the acceptance and expectations of the national health record in Germany, focusing on consumer perspectives, expectations, barriers, information needs, and communication aspects.

**Methods:**

An exploratory, observational, cross-sectional online survey was conducted one year after ePA introduction, followed by descriptive statistical analysis.

**Results:**

The top three ePA use cases were medical document collection, simplified data exchange between medical institutions, and emergency medical information. Adoption barriers included lack of information and access, privacy concerns, and perceived lack of necessity. Participants that felt informed about the ePA, reported they received information primarily from health insurances, the media, and educational or professional sources, mainly through printed brochures, social media, or via emails. Most participants (86.5%) preferred being informed through conversations, particularly with health insurance providers (81.5%) and doctors (61.0%). Written information was highly desired (94.5%), preferably via email or information letters/flyers. However, more than half of the participants (55.6%) reported being uninformed about the ePA introduction.

**Conclusion:**

The study revealed a communication gap between providers and consumers, leading to a low acceptance rate of digital health technologies. Comparisons with other countries showed low adoption rates for opt-in systems. The authors suggest changing communication strategies, given users prefer direct information from doctors or health insurance companies. Adopting an opt-out system with professional social media and marketing campaigns could increase nationwide ePA adoption.

**Supplementary Information:**

The online version contains supplementary material available at 10.1186/s12913-024-12175-6.

## Background

Technological progress drives the introduction of digital health technologies [1]. In Germany, the digitalization of the health and care system is intended to enable citizens to take more self-determined and informed actions [2]. Part of the Ministry of Health's (BMG) national healthcare strategy is to offer various healthcare applications and the secure communication of these services via the so called Telematikinfrastruktur (TI) [2, 3]. The gematik GmbH as the National Agency for Digital Medicine, that is responsible to develop, implement and maintain the TI, it is hold with a 51% stake by the BMG, the remaining shares are distributed among various healthcare organizations of the self-governing body. Therefore, it was entrusted with the conception of this infrastructure and integrated applications. One of the most important applications is the German national health record (EHR), referred to as “elektronische Patientenakte” (ePA) [2].

The ePA was introduced 1^st^ January 2021 in Germany and is available to every person insured under statutory health insurance in Germany, i.e., the predominant form of health insurance that includes 74.3 million people as of April 2024 [4] or approx. 87.8% of the total German population [5]. Healthcare providers and patients can use it to store personal health data in a shared record voluntarily. To this end, they can store documents such as diagnoses, treatment plans, emergency data, certificates of vaccination or a declaration of willingness for organ donation. The consumers decide for themselves which practice, which pharmacy or which hospital may access which health data and for how long. Health insurance funds and statutory health insurers have no access to the stored medical data. This means that data sovereignty always lies with the consumer. Integrated into the German telematic infrastructure (referred to as “TI”), end-to-end encryption is provided. Therefore, special connector hardware is required and installed at the health care service providers [6].

The rapid introduction of various digital health technologies and the system configuration, in which each interest group primarily pursues its own goals, resulted in their uncoordinated implementation. Beyond this, TI-applications were initially introduced partly regardless of their noticeable added value for patients and healthcare professionals [7, 8]. This also applies to the implementation of national electronic health records in Germany [8]. Until the publication of the first national digitization strategy in 2023, various strategies and laws existed for implementing the digitization of the healthcare system; standard guidelines and objectives were lacking [8]. Therefore, the ePA was not widely used.

At the end of 2023, two years after the launch in Germany, ePA adoption rates in different age groups remain low: ~6% (70+y) to 16% (40-49y) [9]. The next step in the strategy published in 2023 was to provide transparent opt-out solutions for the use of the ePA from 2025 [2]. Opt-out means, that every eligible person has a record created unless they object to this.

Against this background, the purpose of the study presented here was to determine the use of the ePA and identify the barriers and facilitators from the consumer's perspective. The study aimed to answer the following research questions:

- What is the ePA adoption rate in Germany?
- What are the expectations of consumers regarding the ePA?
- What are the barriers from the consumer's perspective?
- Which ePA-related information do citizens receive?
- How should communication and information on the ePA be conducted?

### Related work

Recent studies have shed light on various aspects of electronic personal health records (ePHRs) and their impact on patient engagement, data management, and healthcare outcomes. This chapter examines three key studies that provide valuable insights into patient awareness, attitudes, and utilization of ePHRs, as well as their potential benefits and challenges.

#### Patient awareness and preferences

Haug et al. [10] conducted a study focusing on the awareness and attitudes towards the electronic patient record (ePA) among statutory health insurance members in Germany. The study revealed that nearly half of the eligible respondents were unaware of the ePA's existence, indicating a significant gap in public knowledge about this digital health tool. Among those familiar with the system, a majority expressed intention to use it in the future, suggesting a positive outlook once awareness is established. A crucial finding of this study pertains to data transfer preferences. Patients strongly favored having control over their health data, with the most popular option being consent required before each data transfer. This was followed by an opt-out system, while automatic data transfer without restrictions was the least preferred choice. These results underscore the importance of patient autonomy in managing their health information and highlight the need for increased awareness campaigns about the ePA.

#### Patient utilization patterns

Damen et al. [11] conducted a scoping review that provided insights into how patients interact with Personal Electronic Health Records (PEHRs). The study observed that patients often exhibit passive utilization of their PEHRs, particularly when dealing with complex and sensitive medical data. This passive approach manifests as a reluctance to actively generate and manage their own medical information within the system. This behavior stems from concerns regarding the validity, applicability and confidentiality of patient-generated data. Furthermore, it has been noted that patient-generated and -managed health data play a crucial role in ensuring the completeness and currency of medical records, while also being linked to increased levels of patient engagement and satisfaction.

#### Benefits and outcomes of patient-centered digital health records

Brands et al. [12] conducted a systematic review that examined the benefits and outcomes of patient-centered digital health records The review reported high patient satisfaction with these systems. Feasibility (15/19, 97%) and acceptability (23/26, 88%) were positively evaluated. The benefits of patient-centered digital health records were most frequently reported in health care utilization: 77% (10/13) for "use of recommended care services". Benefits of patient-reported outcomes were also mentioned: 70% (7/10) for "disease knowledge", 56% (13/28) for "patient engagement", 56% (10/18) for "treatment adherence", 53% (10/19) for "self-management and self-efficacy". Benefits regarding clinical outcomes were reported: 48% (16/33) for "laboratory parameters" (including HbA1c and LDL). Yet, beneficial effects on "health-related quality of life" were seen in only 27% (4/15) of studies. However, more positive effects were reported, focusing predominantly on active functions. Positive effects were less frequently observed for patients with a high burden of disease and for high-quality studies. Nevertheless, no unfavorable effects were observed, suggesting that patient-centered digital health records have the potential to improve various aspects of patient care and outcomes.

These studies collectively highlight the potential of electronic personal health records to enhance patient engagement, improve healthcare outcomes, and promote patient-centered care. However, they also underscore the need for increased awareness, attention to patient preferences regarding data control, and strategies to encourage active utilization of these digital health tools.

Based on the findings presented in the related work chapter, it is crucial to further investigate the proposed research questions to address significant gaps in our understanding of the electronic patient record (ePA) implementation in Germany and to improve its adoption and effectiveness.

## Methods

We conducted an exploratory, observational, cross-sectional, online questionnaire study to investigate the adoption of and expectations for the ePA.

An interdisciplinary team of experts from the Consumer Health Informatics working group of the *German Association for Medical Informatics, Biometry and Epidemiology* (GMDS e. V.) developed the online questionnaire. The questionnaire was implemented with SoSci Survey in version 3.1.06 [13]. It contains multiple choice questions and scales, which also provides the option “no answer” if participants chose to not answer them. For the original German version and the English translation of the questionnaire, see details in Additional File 1.

The questionnaire was communicated via various channels: (i) Newsletters of professional associations such as GMDS e.V. or of the authors' institutions, (ii) several university mailing lists, and (iii) social media posts via the X/Twitter profile of the working group. Recruitment took place between 03.11.2021 and 02.03.2022.

The results presented here are descriptive, as we refrained from formulating a hypothesis.

### Questionnaire

The questionnaire consisted of 30 questions in 5 blocks that summarized key data about adoption, expectations, barriers, information needs and communication aspects as well as demographic data about the participants.

Since there was a chance that some participants might not be familiar with the ePA, there was an informational text at the beginning of the survey explaining the key aspects about the record (“At a glance: What is the ePA?”). With introductory questions, participants were asked if they know about the ePA and if they are already using it for themselves (dichotomous questions). These questions were necessary requirements to continue the questionnaire and for inclusion in the subsequent analysis.

#### Expectations

Participants were asked what they expect from the ePA in terms of data privacy, data security, and user-friendliness as well as the influence of the ePA on their quality of life and the quality of healthcare ("I expect the ePA to..."). We also asked about accessibility aspects regarding the creation, registration and use of the ePA ("I think that...). Statements were rated on a Likert scale from 1 (=no) to 5 (=fully agree).

Moreover, participants were asked to rate functions according to their usefulness on a *Likert* scale from 1 (=not useful) to 5 (=useful). These included features such as booking tools for doctor’s visits, reminder of appointments, document collection and data exchange between healthcare providers. In addition, participants could enter other functionality they found useful or necessary via an open question format.

#### Barriers

In the section on barriers and obstacles to use, participants who indicated that they did not use the ePA were asked about their reasons. In a half-open question, answer options were given as well as the possibility to give further reasons in a free text field. All participants were asked about their agreement with other statements about the ePA. Topics included concerns about data protection and security, as this is a crucial factor for adoption, according to the literature [14].

#### Information needs

In the survey part on information needs, questions were asked about how participants were informed (e.g. informing people, the form of information). Other questions focused on the individual perception of ePA concepts, and which form and communication channels were preferred for receiving information.

#### Communication aspects

To address communication aspects, we asked related questions about the way of communication and whether the participants feel involved in the implementation of the ePA.

### Statistical analysis

The descriptive statistical analysis was performed using IBM SPSS Statistics version 28 [15].

The R package ggplot2 [16] version 3.4.4 was used for visualizations.

Participants were excluded from the analysis if they did not answer introductory questions which we used for further questions about the knowledge of the ePA and categorization of participants into certain groups e.g. citizens, healthcare professionals and patients and did not answer any other questions.

Missing values were reported but were not included in the calculation of the percentages. If filter questions are used or questions are only presented to certain target groups, the population changes.

Free text was categorized according to the ‘Framework of Nonadoption, Abandonment, Scale-up, Spread and Sustainability’ (NASSS). In 2017, Trisha Greenhalgh et al. presented the NASSS framework, a model that helps to explain non-adoption and abandonment of health technologies by individual users, as well as challenges to the diffusion and sustainability of technology-based change processes in health care institutions (scale-up, spread and sustainability) [17]. The categorization was performed independently by four researchers. To check the interrater reliability, Fleiss’ Kappa [18] was calculated.

## Results

During the accessible period, we observed a total of 2,891 accesses to the questionnaire and 449 questionnaires were started. We excluded 17 participants as they did not fully complete the introductory, basic questions. Since there are numerous aborted questionnaires, we decided to include previously answered questions from these samples. Therefore, sample sizes are specified that are smaller than the number of started questionnaires.

### Demographics

The demographic data showed – in terms of gender – that male (42.4%) and female (37.3%) were evenly distributed. The median age was between 40 and 49 years. 39.4% had an educational level of 6, which meant they have bachelor’s or master’s degree or equivalent, 16.4% even had a doctoral degree. 69.7 percent are insured in a statutory health insurance. Only 18.1% had on average more than one medical treatment or consultation per month. Detailed information is presented in Table 1.

**Table 1:** Cohort demographics, *n*=432

	n	proportion [%]
**Gender**		
Female	161	37.3
Male	183	42.4
Gender-diverse	3	0.7
Prefer not to answer	9	2.1
No response	76	17.6
**Age**		
18–29	55	12.7
30–39	66	15.3
40–49	64	14.8
50–59	91	21.1
60–69	42	9.7
70–79	29	6.7
>= 80	6	1.4
Prefer not to answer	3	0.7
No response	76	17.6
**Education level (acc. to ISCED 2011, see Additional file 2)**		
Lvl 2 Lower secondary education	24	5.6
Lvl 3 Upper secondary education	67	15.5
Lvl 4 Post-secondary, non-tertiary education	0	0.0
Lvl 5 Short-cycle tertiary education	17	3.9
Lvl 6+7 Bachelor, Master or equivalent	170	39.4
Lvl 8 Doctoral or equivalent	71	16.4
No qualification or prefer not to answer	7	1.6
No response	76	17.6
**Occupation**		
Employed	236	54.6
In Professional training	16	3.7
Retired	48	11.1
Unable to work	5	1.2
Self-employed / freelancer	20	4.6
Civil servant	16	3.7
Other	8	1.9
Prefer not to answer	7	1.6
No response	76	17.6
**Medical Professional**		
Yes	117	27.1
No	315	72.9
**Status of Health Insurance**		
Statutory health insurance	301	69.7
Private health insurance	47	10.9
Prefer not to answer	8	1.9
No response	76	17.6
**On average more than one medical treatment or consultation per month**		
Yes	78	18.1
No	354	81.9
**Number of known illnesses**		
None	131	30.3
1	77	17.8
2	61	14.1
3	37	8.6
>=4	34	7.9
Prefer not to answer	16	3.7
No response	76	17.6

Four hundred three participants were aware of the ePA, 93 participants used an ePA, 309 did not use it, 30 did not answer this question (total of *N*=432 participants).

### Expectations

Participants scored 19 use cases or potential functions of the ePA on a 5-point Likert scale from not useful (1 point) to useful (5 points). 344 participants answered this question. Participants rank the ePA as a collection of medical documents (xrays, Maternaty log), vaccination certificates, etc. as most useful (mean = 4.7, sd = 0.86).

**Fig. 1:**
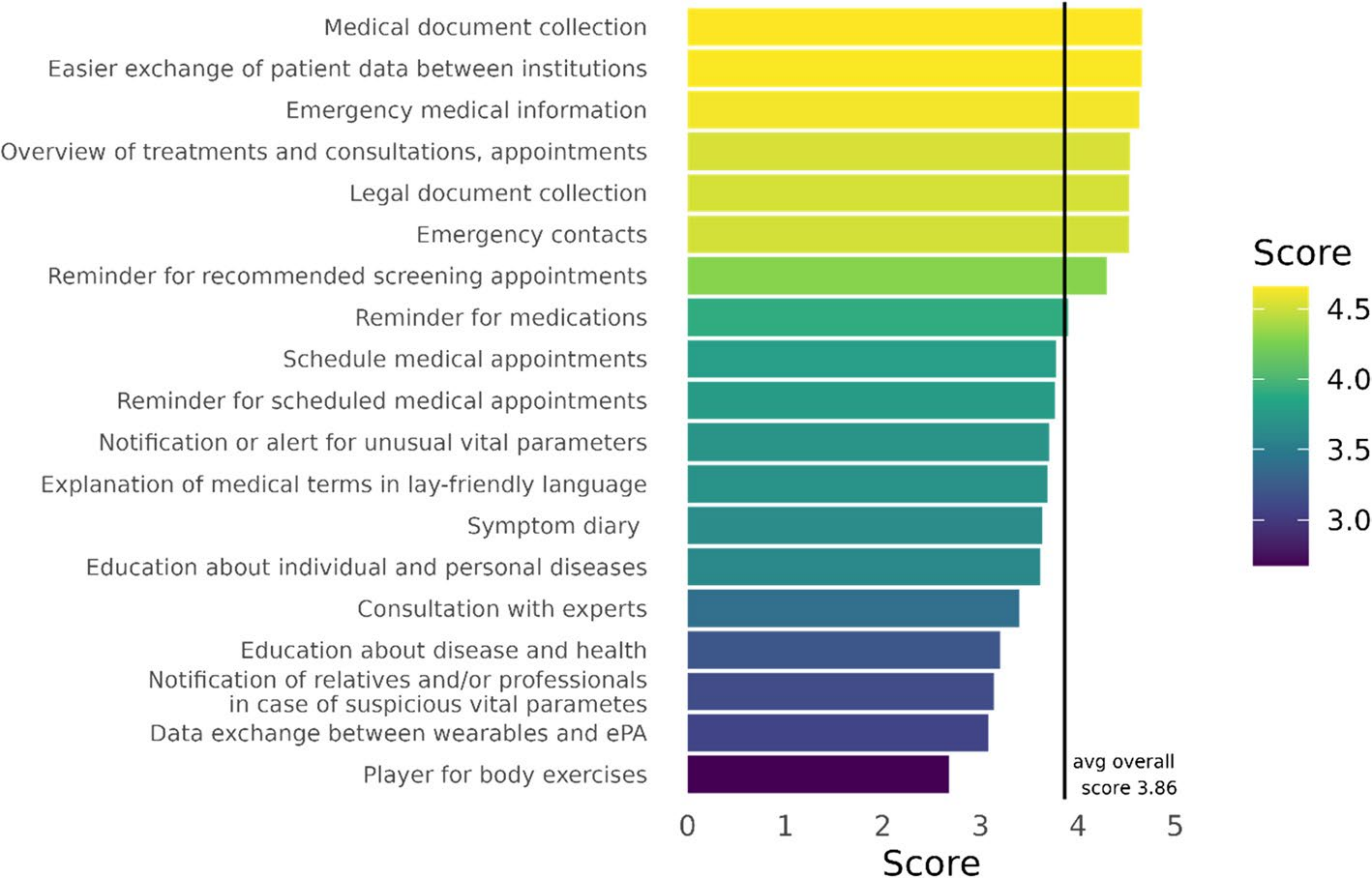
Functional usefulness for 19 ePA use cases (Likert scale: 1–5); *n*=361

The top 3 scored use cases are (1) medical document collection, (2) simplified exchange of patient data between medical institutions and (3) emergency medical information (allergies, medication, etc.). Figure 1 shows the use cases described as beneficial by the participants.

**Fig. 2:**
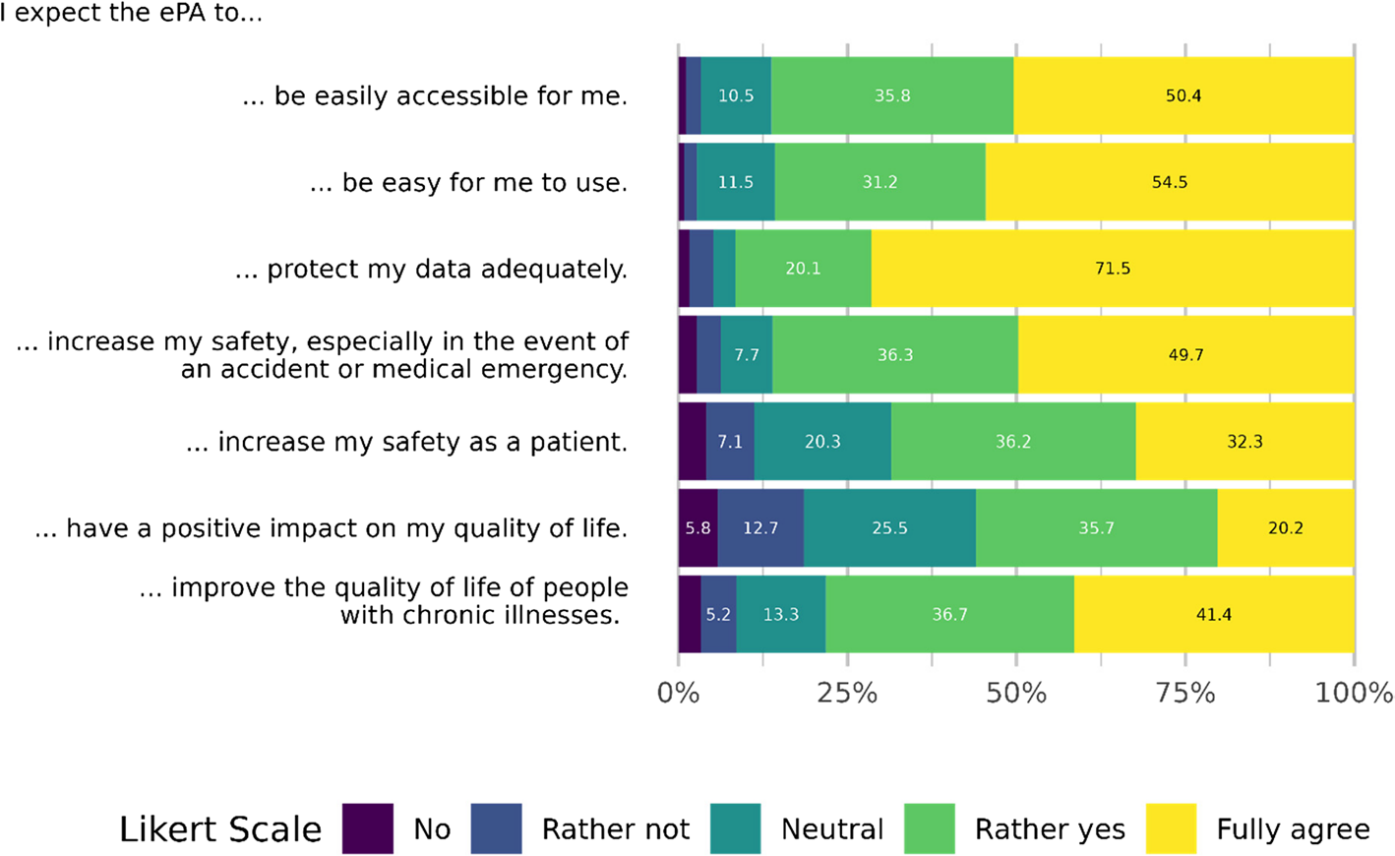
Expectations regarding the ePA

The participants were asked to rate their expectations concerning the ePA from 1 (=no) to 5 (=fully agree); Figure 2 shows the distribution. Overall, expectations were high. The median is 5 (=fully agree) for accessibility, user-friendliness, data privacy and increased safety in the event of accidents or medical emergencies. Expectations are somewhat lower for the topics of patient safety, quality of life and improving the quality of life of chronically ill patients (Md=4, rather yes).

**Fig. 3:**
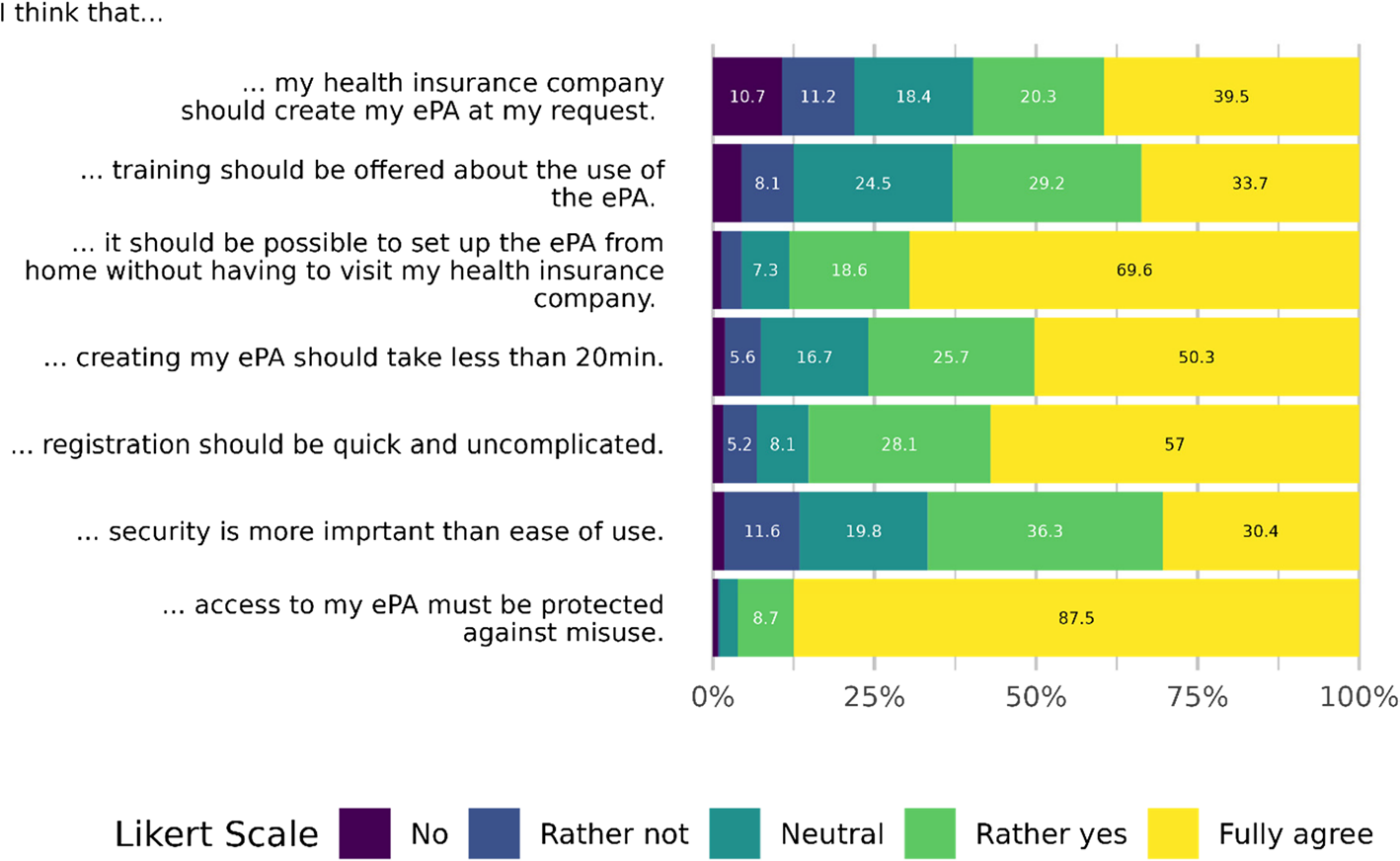
Views towards using the German ePA

We asked the participants about their specific views ("I think...”) on using the German ePA. Figure 3 shows that expectations are high. The participants expressed that (a) their health insurers create the ePA, that (b) training is offered, (c) access should be possible from home, (d) the creation of the ePA should take less than 20 minutes and that (e) the registration process is quick and uncomplicated. Security seemed more important than user-friendliness. Almost all participants fully agreed that access to an individual’s health record must be protected against misuse.

### Barriers

**Fig. 4:**
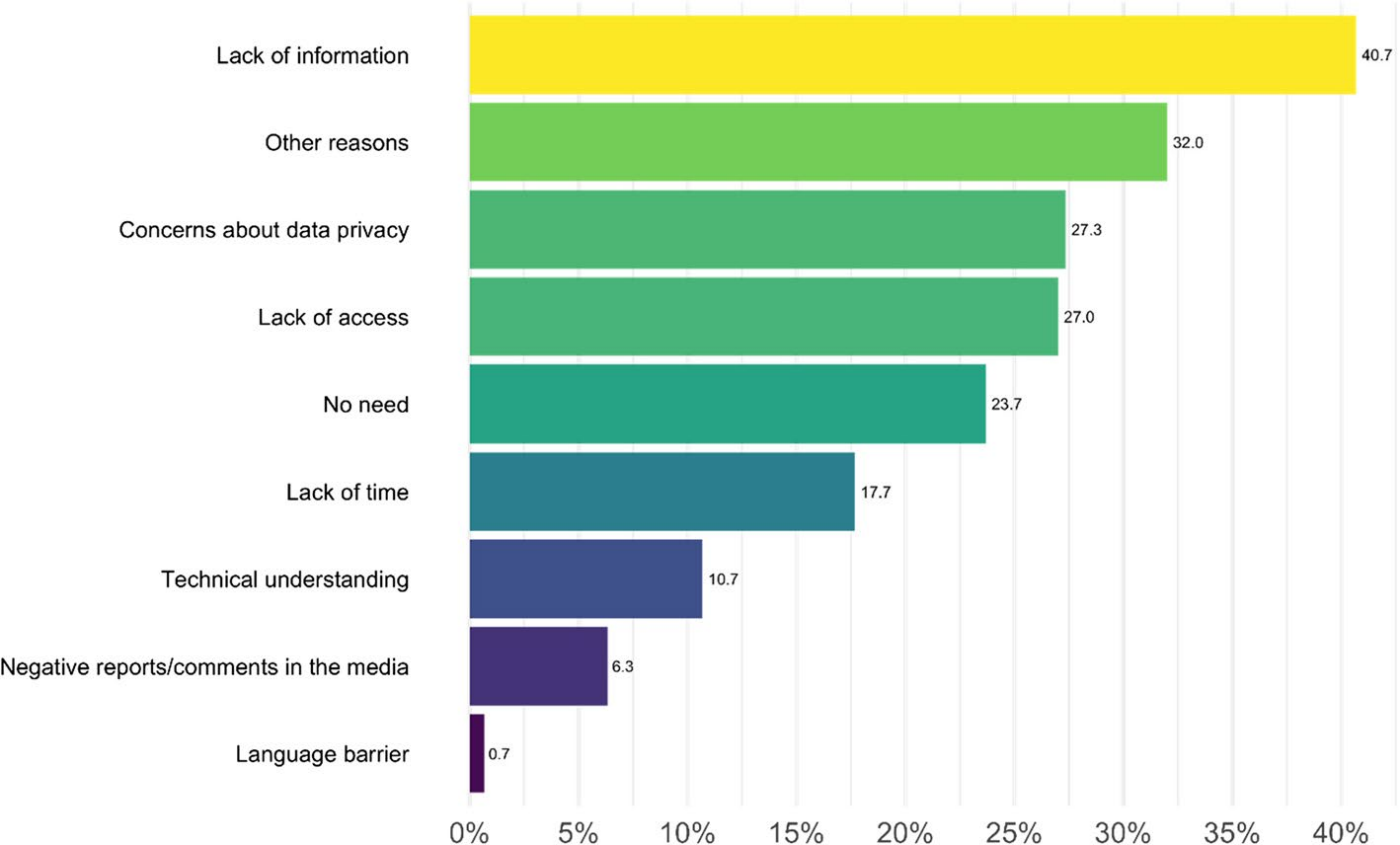
Reasons of non-use for participants (*n*=300); multiple answers could be given

Three hundred participants answered this question and gave a total of 558 answers. As shown in Fig. 4, 40.7% of participants (122/300) reported that they do not use the ePA because they lack information about it. 81 participants stated the lack of access and 82 concerns about privacy. 71 participants did not see the necessity to use the ePA. 53 participants stated that they do not have the time to use the ePA, 32 reported lack of technical understanding and 19 did not use it because of negative reports or comments in the media. Only two participants stated that there are language barriers, 96 mentioned other reasons.

Participants provided free-text entries for “other reasons” why they do not use the ePA. These entries were coded in the next step: There was a total of 115 codings in the dimension “Technology”. Most of them were in the subdimension “material features” (*n*=59). Therefore, contributions to complexity in this domain resulted first from the quality characteristics of the technical systems (e.g. usability, robustness, maintenance efforts). In addition, there were codings in the sub-dimensions, which referred to data types (*n*=13), the knowledge of use (*n*=40) and the dissemination of technology (*n*=3).

There was a total of 121 codings in the dimension “Adopter”. Most of them were in the subdimension, which concerned the role of the patient (*n*=76). It addressed the adoption by patients or clients, including acceptance (hence symbolic meaning and aesthetics) and the work required by them. Complexity arose from inapplicable assumptions regarding the actors, such as health literacy and patient ownership [16]. 45 codings were associated to the role of employees. A major significance for complexity in this domain addressed the acceptance of the change, e.g. if the change conflicted with the professional self-image of the professionals or strongly changes established roles and professional practices.

In total 57 codings in the dimension “organization” were recorded. The domain included contributions to complexity that arose at the level of the institution(s) of health care in which the technology is used. Fifty-three of the codings were assigned to the sub-dimension “Readiness for this technology/change”. Three codings were assigned to the sub-dimension “Capacity to innovate” and one coding to the sub-dimension “Nature of adoption/funding decision”.

The dimension “Wider System” comprised 54 codings, 24 codings concerned the role of policy. In general, technology-based changes in health care institutions are of course also highly dependent on the framework conditions of the respective health care system (macro level). There were 8 codings in the dimension “Wider System”, which concerned the role of the regulatory/legal framework. Influences on complexity have legal framework conditions (e.g. data protection). 16 codings concerned the role of professional, and six sociocultural perspectives. The socio-cultural framework conditions included, for example, attitudes of the general population.

Only eight codings were assigned to the dimension “Condition”. Three codings to the area “Nature of condition or illness”, five to “Comorbidities, sociocultural influences”.

To assess the extent of the matches, the interrater reliability was calculated via Fleiss' Kappa. For the coding of *other reasons (for non-use)* using the NASSS framework, a strength of agreement of “slight” (.172) was achieved.

Participants were also asked how well they felt involved in the introduction of the ePA. For this purpose, the sample was divided into patients (in treatment more than once a month on average, *n*=54), citizens (*n*=295) and professionals (*n*=78).

**Fig. 5:**
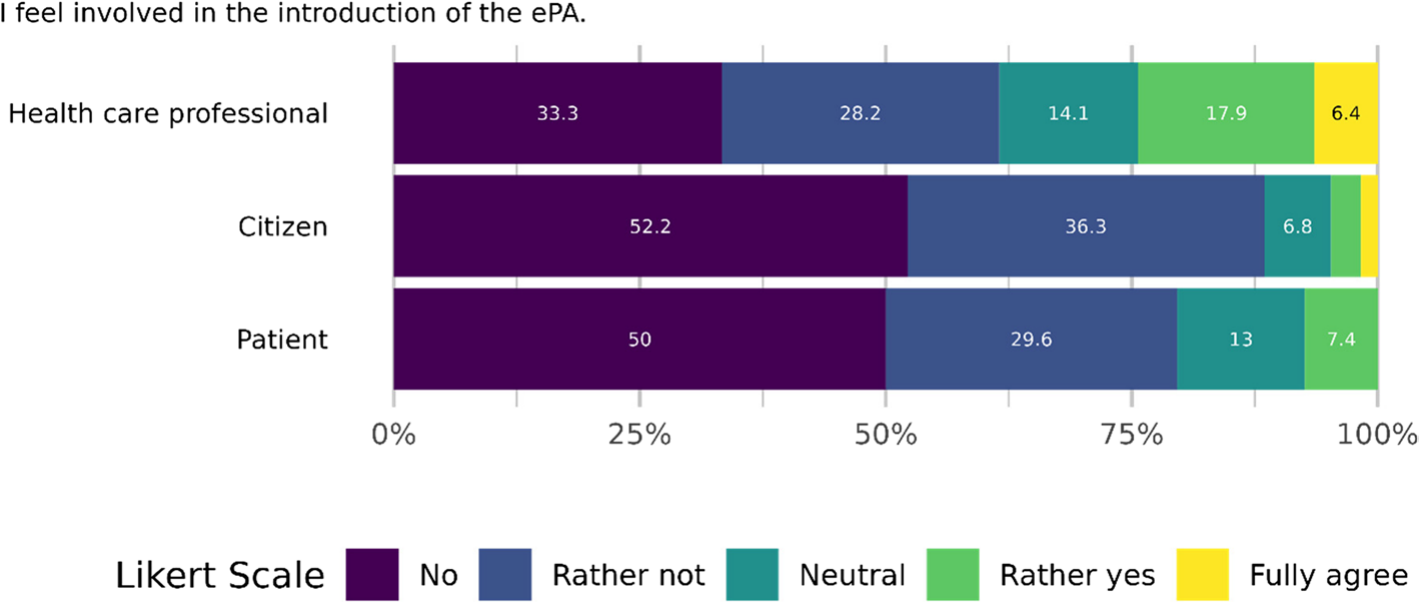
Involvement in ePA introduction per user group (5-point Likert Scale: 1=no, 5=fully agree)

Figure 5 shows the distribution of perceived involvement in the three groups. The median in the specialist group is 2, which means that they tend not to feel involved. The median for the patient group is between 1 and 2, meaning that they also feel little to no involvement. The lowest median (=1) is for the Citizen group, which means that they do not feel involved at all.

### Information Needs

The participants were asked in which form they would like to be briefed to feel sufficiently well informed. 356 participants answered the question. 308 out of 356 (86.5%) would like to be informed in a conversation. Of those participants 81.5% (251) said they would like to have this conversation with the health insurance, 61.0% (188) mentioned the doctor. Only a few participants wanted conversation with the employer (20; 6.5%), in education or training (23; 7.5%), with family members (16; 5.2%) or with friends/acquaintances (17; 5.5%).

In a free text field the participants stated, that they preferred to be informed by a data protection officer, IT consultants, independent organizations, via services of the federal ministry of health (BMG) or by gematik GmbH, Case managers, Ministry of Health, state education agencies, trained medical professionals at health care providers (doctor's office, hospital, etc.), caregivers, through participants own enquiries or by public media.

326 out of 356 (94,5%) participants would like to be informed in writing. About two thirds of the participants (235/326; 72.1%) would like to receive the written information via e-mail, 60.1% (196/326) via an information letter or flyer.

261 out of 356 participants would like to be informed digitally. Of these, about two thirds (175; 67.0%) say they would like to be informed via their health insurance application. About one third of the participants (93; 35.6%) via a social media campaign, about a half (124; 47.5%) via a multimedia offer such as videos.

240 out of 356 (67.4%) participants would like to be informed through traditional media offers such as newspapers (160; 66.7%), television (143; 59.6%) or radio (102; 42.5%). Only 87 out of 240 (36.3%) would like to be informed via podcasts. Further wishes from free text forms were public awareness campaigns and journals of health insurances.

### Communication aspects

More than half of the participants report that they were not informed about the introduction of the ePA (240, 55.6%). 118 (27.3%) participants were informed about the ePA, while 74 (17.1%) did not answer this question (total of 432 participants).

About one half of the informed participants (60/118, 50.9%) were informed by their social health insurances about the ePA. 38.1% (45/118) were informed by the media and 28.0% (33/117) were informed about the ePA as part of their education or professional qualification. In contrast only 4.2% (5/118) were informed by their doctors. In the free text field, the participants also mentioned that they were informed due to a professional background /study, a health insurance company, specialist portals or directly via gematik GmbH.

When asked how they were informed, most participants said they were informed through printed brochures (44/118, 37.3%), followed by social media (29/118, 24.4%) and emails (25/118, 21.0%). In addition, participants were asked how well informed they felt about the introduction of the ePA, rated on a Likert scale. For this purpose, the sample was divided into patients (in treatment more than once a month on average, *n*=54), citizens (*n*=301) and professionals (*n*=79).

**Fig. 6:**
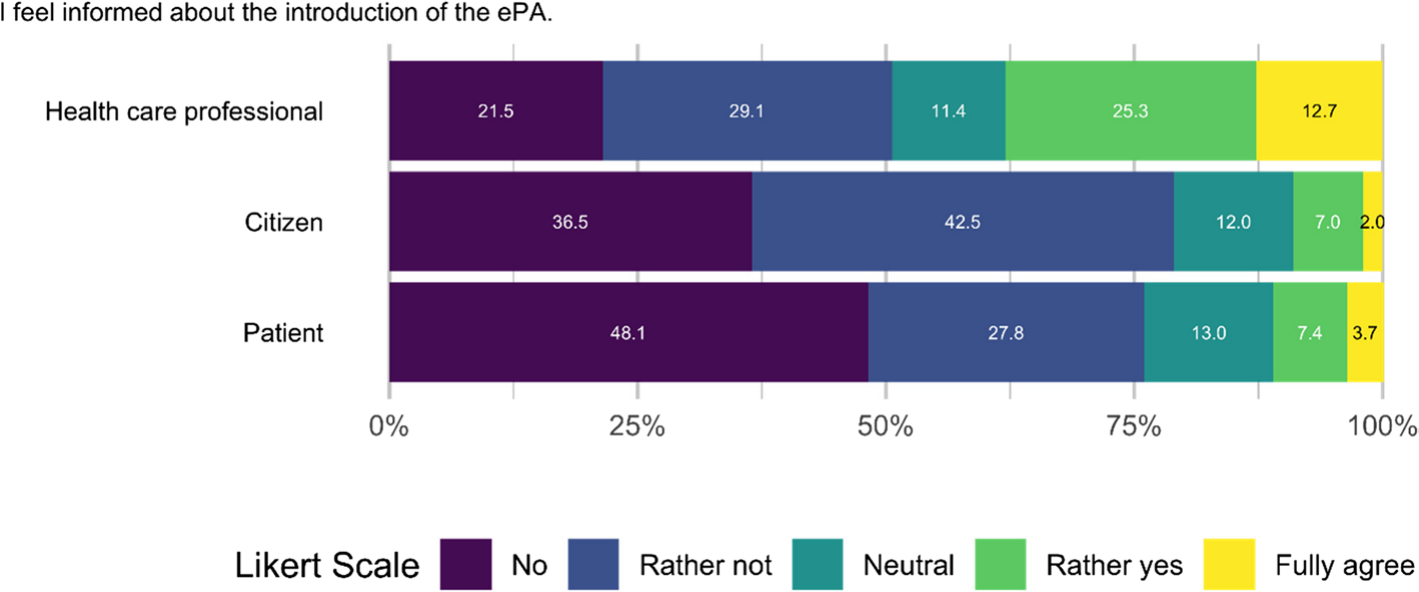
Information on ePA introduction per user group (5-point Likert Scale: 1=no, 5=fully agree)

Figure 6 depicts the distribution of the perceived level of information in the three groups. The median value for all groups is two, which means they felt rather not informed.

However, the 25th and 75th percentiles revealed some differences between the groups. At the 25th percentile, both patients and citizens reported a score of 1.0 (=no), indicating that at least 25% of respondents in these groups felt minimally informed. In contrast, professionals showed a slightly higher level of perceived information at this percentile, with a score of 2.0 (=rather not). The 75th percentile scores highlighted more pronounced differences. While citizens maintained a score of 2.0, patients showed a slight increase to 2.25. Notably, professionals demonstrated the widest range of responses, with a 75th percentile score of 4.0 (=rather yes), suggesting that a quarter of the professionals felt considerably more informed than the other groups.

Participants were also asked with whom they had spoken to about the ePA. 192 of 432 participants (44.4%) responded, 158 of 432 participants (36.6%) stated they talked but with no group or person that were provided in the answering options, 82 of 432 did not answer (19.0%). Around 56% each said that they had spoken to family members (109/192) and work colleagues (108/192). Almost as many (104/192, 54.2%) said acquaintances/friends. 30.2% mentioned doctors (58/192) and 25.5% (49/192) the health insurance company. The free text field also included: Professors/Teachers, Science journalist, Politics, associations, IT company, Family practice staff or/and professional societies.

## Discussion

The results of the survey showed that the ePA was and is still not widespread. This is confirmed by a recent survey in Germany of Kühnel et al. [9]. Our results show that one year after the introduction of the ePA, 21.5% of our respondents were using the ePA and 93.3% were aware of it. Furthermore, they report that 9% of the German population use the ePA and 76% have heard of it (as of December 2023 - two years after our survey). This comparatively high number in our results might stem from a high level of education of the participants on average, as well as the recruitment process, which targeted health-related associations, study programs, etc. (see Limitations).

De facto non-use in Germany is not necessarily to be understood as active rejection but is often due to insufficient knowledge on the part of patients and providers and the existing legal framework [19]. This is in line with the observation of this study as only 27.3% of the participants stated that they had been informed about the ePA. Furthermore, the results show the request for information via channels such as email, social media, newspapers, television, etc.

The top three scored use cases in our study are (1) medical document collection, (2) easier exchange of patient data between medical institutions and (3) emergency medical information (allergies, medication, etc.). Similar functions included in patient health records were identified in the systematic review by Harahap et al. [20]. The authors found more than 50 studies that reported use cases comparable to (1) and (2). The management of emergency medical information seems to be not the focus of the studies. However, medication and prescription management as an “Advanced function” of a patient health record is reported by numerous systems [20].

Interestingly, already in 2012, Kharrazi et al. performed an evaluation of nineteen “stand-alone mPHR applications" and regarded emergency contact information as one of the most important data elements to be included in a patient health record [21]. Overall, the expected and as most-useful perceived use cases by the participants match the provided functions of the German ePA as well as many other EHRs and PHRs. One drawback of the review by Harahap is that it included several approaches of electronic health records as well as methodologies, e.g., stakeholder interviews prior launch, conceptual papers and regional projects.

The features that consumers perceived as useful in this study surpass those currently offered by the ePA, e.g., “Schedule of appointments” and “Reminder for scheduled medical appointments”. However, our findings indicate that participants wished for the ePA to become a central hub that helps them to navigate their patient journey and complex treatment scenarios. This vision aligns with a whitepaper from 2020 issued by gematik GmbH [22]: Providing an infrastructure with the TI as backbone with interfaces to third-party apps. In this vision of a digital healthcare system, an ecosystem of medical application from primary and secondary market could be integrated seamlessly. To achieve such a vision, a strong political will is needed to unite all key stakeholders.

Observing the transformation process of the German healthcare system, political will seems to be quite selective. Here, it is worthwhile to compare the introduction of nation-wide Digital Health Applications (DiGA) in 2020 with the ePA introduction. DiGAs are certified and evaluated applications, e.g., smartphone apps, that can be prescribed like an ordinary drug [23]. Since 2016, the BMG commissioned evaluations on how to integrate innovative mobile health applications in routine care, i.e., roughly four years were needed to get medical mobile applications into the complex German healthcare and reimbursement system. For the ePA and its predecessors, the design and development phases now span more than two decades. In view of the DiGAs’ introduction, it can be concluded that there were more substantial political efforts for a rapid distribution than currently observed for the ePA.

Initiatives like Digital Navigators (DN) [24] were established to boost DiGA acceptance and usage. The DigiNavi study pilots DN in primary care and outpatient psychiatric services, indicating strong political support for DiGA. In comparison DiGA implementation appears more targeted and user-oriented than ePA. While ePA faces challenges in standardization, security, and user-friendliness, DiGA sees concrete measures for increasing acceptance and competence development. ePA implementation struggles with interoperability and governance structures. At the same time, it is easier to implement a "single" application such as a DiGA with low connection to other Healthcare-IT-Systems to date, than a more complex product such as the ePA.

In view of the planned implementation of the opt-out procedure in Germany in 2025, the question arises as to how communication can accompany a "new start" for the ePA. Various studies have focused on the communicative elements of ePA use, but most of them focus on communication between doctor and patient in the immediate treatment context or on the effects of EHR use on the doctor-patient relationship [25, 26]. To improve ePA adoption, also a multi-channel approach should be implemented, focusing on clear, comprehensive information, especially also about beneficiaries and the management of potential risks of use, disseminated through health insurance providers, healthcare professionals and various media platforms. Special attention should be given to addressing the current lack of information and ensuring that all demographic groups are adequately informed.

### Limitations

To better contextualize the results in the following comparison, further limitations regarding the population reached should be mentioned. It is possible that the procedure for recruiting participants led to a selection bias. In this context, most participants have a high level of education. This may not be representative of the overall population. Furthermore, it is possible that mainly people with a high or very high affinity for information technology have answered the questions respectively answered the questionnaire. Due to the relatively high percentage of people who have given no information at all on their state of insurance, it might well be that possibly the percentage of privately insured respondents of this study exceeds the share of the total population. To estimate the extent of a potential selection bias, the authors tried to compare participants’ data with the last survey of educational qualifications by the Federal Statistical Office [27]. Unfortunately, this is not directly possible due to different survey methods. Here, a distinction is made between the highest school-leaving qualification and the highest vocational qualification, while in the survey both were integrated into the overall highest educational qualification. Nevertheless, as expected, the proportion of people with higher educational qualifications in the overall population is lower than in the sample reached. The aspect of ethnic groups and language skills was not part of the compilation of the sociodemographic data. Here it is also quite possible, that mainly native German speakers respectively people with very good German skills have participated in the survey. This means that representativeness is not given and language barriers may constitute a general obstacle regarding the self-determined use of the ePA, even a migratory background does not per se go along with a lower digital health literacy [28]. Since there are different concepts of electronic records in the healthcare system, the authors had in advance integrated a short explaining text for the participants of the survey to reach a coherent term understanding. Nonetheless it is of course possible that against the background of different experiences with digital health-related applications that the participants of the survey did not always just follow the concept of the German ePA that was presented here.

### Comparison with prior work

When it comes to the acceptance and increased uptake of eHealth in Germany, decision-makers, study authors, etc. often say that patients and service providers need to be "taken on board” – without explaining this sentence in more detail. It is therefore worth looking at what other countries are doing to increase the uptake of ePA. For reasons of comparability with the results of this survey, only electronic health records like the nationwide German ePA will be discussed in the following paragraphs.

An increasing number of citizens in European and other countries has access to a personalized health record, such as the German ePA. However, access and use (e.g. secondary use in health research) are regulated differently [29, 30]. For example, countries such as Austria and the Scandinavian countries chose an opt-out approach [31]. A study conducted in 2021 on the legal interpretation of the ePrivacy Directive [32] showed that the opt-out procedure leads to significantly higher usage rates. In Austria, for example, 97% of the population use an ePA. In Denmark, where ePA has been mandatory since 2004, almost all General Practitioners (GPs) and pharmacies, hospitals and specialists use electronic records [29].

The national personal health record in Australia was introduced in 2012. The initial adoption of the system was considered low with only 21% as of 2017 [33]. After changes in legislation the original opt-in model was changed to an opt-out model in 2018 to increase the adoption. The ePA in Germany was also launched as opt-in and will be transferred to an opt-out model in 2025.

The Australian Digital Health Agency reports on the Implementation of the *My Health Record* system in 2019 [34]. They stated that the communication strategies were effectively implemented. In 2017, a communication plan was crafted based on evaluations from earlier participation trials and additional market research. This plan involved creating, testing and distributing various informational materials such as brochures, posters, and videos targeted at specific audiences. The Australian Digital Health Agency (ADHA) monitored the reach of these communications, finding that the average Australian was exposed to information about My Health Record approximately 38 times during the opt-out period, leading to a rise in public awareness over time. Additionally, ADHA conducted educational initiatives for healthcare providers. These efforts ensured that every general practice and community pharmacy in Australia had access to educational resources about *My Health Record*, which was reflected in the increased registration and usage of the system.

Pang and Chang [35] analyzed Social Media posts on Twitter and suggested communicating with stakeholders via social media as well. This matches our study’s findings, with over one third of the participants stating they wanted to be informed via a social media campaign. The literature research does not provide explicit details on the methods by which citizens are generally informed about the existence of a health record. Only about France is it described that the first information was received from the social security organization via E-Mail. After that the physicians constantly hand out flyers to their patients [36].

Besides the information about the results, there is follow-up information, which also should be communicated. Abd-Alrazaq et al. conclude that “promotional campaigns about functions and features of the system” should be conducted whereas direct communication between patient and health care professional could be one of the most effective channels [37]. The results of our survey support this: Almost 90% of the participants preferred face-to-face information.

In addition to the problem of communication regarding the release of the ePA, we observed barriers in our study. Yousef et al. [38], also observed some barriers regarding the personal health record adoption in Saudi Arabia from the perspectives of healthcare providers (HCP). The top 3 barriers to Nationals Electronic Health Record adoption were lack of patient awareness, patient low literacy and patient resistance to new technologies. In a qualitative approach three primary themes were identified in the comments of the survey: general perceptions of the Nationals Electronic Health Record (positive attitudes, negative attitudes, additional features), 2) patient engagement as a requirement for the successful implementation of the Nationals Electronic Health Record and 3) education/training of HCPs, patients, and caregivers. When comparing our results, there is a certain overlap. Barriers mentioned such as lack of access or time could be interpreted as a lack of engagement in the acquisition. A lack of technical understanding, on the other hand, could be overcome through the training of patients.

An additional barrier which is stated in our results is the concern about data protection. 14.7% of the non-users stated that they have concerns about data protection (top 3 reason for non-use). This is in line with the results of the national EHR in the UK. The National Health Service (NHS) introduced an England-wide ePHRs in 2015. In the years after launch the adoption remained low with 24.4% in 2018 [37]. In their study based on the unified theory of acceptance and use of technology (UTAUT 2.0) framework, they found that patients are more likely to use ePHRs if it is perceived as „very useful and advantageous“ [37]. Moreover, patients are more likely to use an ePHR if it is secure and maintains data privacy. The healthcare system in France follows another approach regarding data-protection. There the EHR is an optional and voluntary process for patients, regulated by the Act of Public Health Code. Patients create and manage their EHR, accessible online for medical monitoring. Access rights are stringent, with only attending physicians and Emergency Medical Services having full access. It could be expected that there will be further discussion on data protection and privacy. In Australia, the change to the opt-out strategy led to “vociferous discussions of the privacy concerns” [39] and privacy is considered a major issue in Germany as well.

## Conclusion

Given the results of this study, health policy in Germany requires changes in ePA communication. Low adoption rates and lack of awareness among the population indicate that existing communication strategies have been insufficient, as adoption is associated with perception of benefits (cf. [37, 38]). Consumers expect the ePA to be a secure, accessible and user-friendly system that facilitates the collection and exchange of medical information, with a strong emphasis on data privacy and improved healthcare outcomes. Yet, several barriers hinder ePA adoption. The primary obstacle is a lack of information. Other significant barriers include privacy concerns, lack of access, perceived lack of necessity, time constraints and insufficient technical understanding. Further challenges include technical issues, complexity arising from health literacy assumptions, and readiness for technological change.

The study also revealed a low level of perceived involvement in the ePA introduction across patients, citizens and professionals. Most participants reported feeling uninformed about the ePA, with over half stating they received no information about its introduction. Those who were informed primarily received information from health insurance companies, media sources, or through their education or professional qualifications. Even with a sample that is biased towards higher education qualifications and high IT affinity, this study sheds light on the early adopters of ePA with their expectations and barriers they faced in 2021 and 2022. Especially regarding the opt-out transformation in early 2025, the presented findings provide valuable ground for future surveys.

An uncoordinated introduction of digital health technologies leads to a low acceptance of the ePA. The results of this study show a communication gap between providers and consumers. However, a comparison with other countries shows that this is not only a problem of German implementation. It appears that other countries with opt-in procedures have comparable low adoption rates. One solution to the problem appears to be a change in communication strategy. Users want direct information from doctors or health insurance companies. The example of the Australian strategy shows that the change to an opt-out solution in conjunction with a professional social media and marketing campaign as well as educational offers could increase the adoption of the ePA.

## Supplementary Information

Additional file 1. Original Questionnaire (English version). The translated version of the original questionnaire is provided as a supplemental PDF-File (ePA-Survey-Questionnaire-english.pdf).

Additional file 2. Mapping of German Education Levels to International Standard Classification of Education (ISCED).

## Data Availability

The datasets used and/or analyzed during the current study are available from the corresponding author on reasonable request.

## Abbreviations

BMG: German Federal Ministry of Health
DiGA: Digital Healthcare Application
eIDAS: Regulation on Electronic Identification and Trust Services
ePA: Electronic Health Record (German: elektronische Patientenakte)
gematik: Gesellschaft für Telematikanwendungen der Gesundheitskarte
GKV: Statutory health insurance (German: Gesetzliche Krankenversicherung)
GP: General Practitioner
HCP: Health Care Provider
IT: Information Technology
NHS: National Health Service (UK)
PEHR: Personal Electronic Health Record
PHS: Public Health Service
SGB: German Social Code
TI: Telematics Infrastructure
