# Supplementary material for: Consumer perspectives on the national electronic health record and barriers to its adoption in Germany: does health policy require a change in communication?
Source: BMC Health Serv Res. 2025 Jan 7;25:33. doi: 10.1186/s12913-024-12175-6 (PMC11706193; doi:10.1186/s12913-024-12175-6)
Supplement: Supplementary file 1 — Additional file 1. Original Questionnaire (English version). The translated version of the original questionnaire is provided as a supplemental PDF-File (ePA-Survey-Questionnaire-english.pdf). [file 12913_2024_12175_MOESM1_ESM.pdf]

Page 01

IN01

1. Welcome to

Dear participants,

The Consumer Health Informatics (CHI) working group of the GMDS (Society for Medical Informatics, Biometry and Epidemiology) is conducting a **survey on the use of electronic patient records (EPR).**

The aim of this survey is to gather your expectations and opinions on the opportunities and barriers to implementation of electronic patient records. Every completed questionnaire helps us to do this. We therefore kindly ask you to complete the questionnaire in full. Participation in the survey is
voluntarily. Of course, we assure you that we will comply with all statutory data protection regulations. Your answers will only be analyzed anonymously. No reference to your person can be made at any time.

We will also be happy to provide you with the evaluation results afterwards. If you are interested in the results, please enter your e-mail address at the end. We will then be happy to send you the results. If you have any questions or would like further information, please do not hesitate to contact us by e-mail at ag.chi@gmds.de.

It will take about 15 minutes to complete the questionnaire. Please only take part in

the survey if you are at least 18 years old.

What should I do?

Please read each statement carefully. For each question, decide how much the statement applies to you and select the option that suits you best. For some questions, you can choose multiple answers or enter free text. If this is the case, a note is inserted there. Please avoid free text answers that could reveal your identity.

Please do not skip any answers and answer spontaneously. If you have difficulty answering a statement, choose the answer option that best applies to you.

If you are participating via a smartphone, we recommend that you use it in landscape format. Note:

There are no right or wrong answers.

Thank you for your support in this project!

Auf einen Blick: Was ist die elektronische Patientenakte (ePA)?

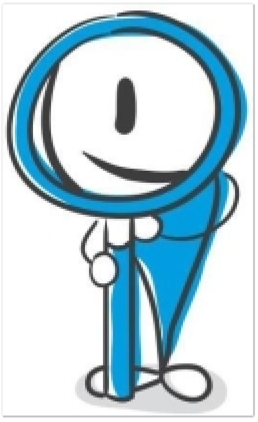

Bild: Strichfiguren.de; AdobeStock

- Die ePA stellt eine digitale Sammlung von medizinischen Informationen und Dokumenten dar. Das bedeutet, dass alle für die Behandlung notwendigen Informationen vom PC, Laptop oder über das Smartphone abrufbar sind.
- In der ePA können bspw. Röntgenbilder, Arztbriefe, Entlassbriefe, die Patientenverfügung, Notfalldaten und Laborergebnisse gespeichert werden.
- Der Zugriff auf die ePA kann von verschiedenen Orten erfolgen. Es ist mit der ePA nicht mehr notwendig, zu Behandlungsterminen alle medizinischen Unterlagen mitzunehmen. Alle Dokumente sind ortsunabhängig verfügbar.
- Wesentliche Ziele der ePA sind, dass alle wichtigen medizinische Informationen rasch verfügbar sind, Patienten*innen einen besseren Einblick in das Behandlungsgeschehen erhalten und unnötige Doppeluntersuchungen möglichst vermieden werden.
- Die Nutzung einer ePA ist freiwillig. Nur die Patientin/der Patient kann entscheiden, dass auch andere Zugriff auf ihre/seine Daten und medizinischen Dokumente haben.
- Die ePA unterliegt hohen Anforderungen an Datenschutz und Datensicherheit. Alle verwendeten Systeme sind von der zuständigen Behörde überprüft und zertifiziert.

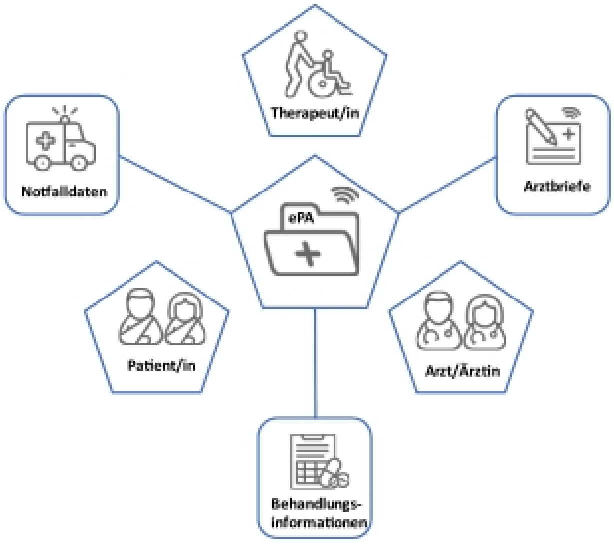

Das Prinzip der Elektronischen Patientenakte (Bild: ZTG GmbH)

Page 03

IN03

2. Are you currently working in a medical specialty?

In this survey, we include: doctors, nurses, medical documentalists, medical assistants and other healthcare professions (physiotherapy, speech therapy, etc.) and administrative staff.

○ Yes ○ No

2 active filter(s)

Filter IN03/F1
If one of the following answer option(s) was selected: **1**
Then show question/text **EB05** later in the questionnaire (otherwise hide)

Filter IN03/F2
If one of the following answer option(s) was selected: **1**
Then show items **5,6** in question **EV06** (otherwise hide)

Page 04

IN07

3. Do you receive treatment more often than once a month on average (e.g. in a doctor's practice, in a clinic, as part of a rehabilitation or physiotherapy program, etc.)?

○ Yes ○ No

8 active filter(s)

Filter IN07/F1
If one of the following answer option(s) was selected: **1**
Then show items **1** in question **EV06** (otherwise hide)

Filter IN07/F2
If one of the following answer option(s) was selected: **1**
Then show items **3** in question **EV06** (otherwise hide)

Filter IN07/F3
If one of the following answer option(s) was selected: **1**
Then hide items **2** in question **EV06**

Filter IN07/F4
If one of the following answer option(s) was selected: **1**
Then hide items **4** in question **EV06**

Filter IN07/F5
If one of the following answer option(s) was selected: **2**
Then hide items **1** in question **EV06**

Filter IN07/F6
If one of the following answer option(s) was selected: **2**
Then hide items **3** in question **EV06**

Filter IN07/F7
If one of the following answer option(s) was selected: **2**
Then show items **2** in question **EV06** (otherwise hide)

Filter IN07/F8
If one of the following answer option(s) was selected: **2**
Then show items **4** in question **EV06** (otherwise hide)

Page 05

4. Have you ever heard of the electronic patient record (EPR)?

IN04

○ Yes ○ No

1 active filter(s)

Filter IN04/F1
If one of the following answer option(s) was selected: **1**
Then show question/text **IN05** later in the questionnaire (otherwise hide)

Page 06

5. Do you already use the electronic patient record (EPR) as part of your healthcare?

IN05

○ Yes ○ No

1 active filter(s)

Filter IN05/F1
If one of the following answer option(s) was selected: **1**
Then hide question/text **BH02** later in the questionnaire

Page 07

BH01

Barriers and obstacles to use

The following section deals with barriers and obstacles to using the electronic patient record (EPR).

BH02

6. If you have decided not to use an ePA so far: What are the reasons for this?

Multiple answers possible

- [ ] Technical understanding
- [ ] Lack of access
- [ ] Concerns about data protection [ ]
Linguistic barriers
- [ ] Missing information
- [ ] Negative reports/comments in the media [ ] No
need
- [ ] Lack of time Other
- [ ] reason ______

- [ ] No answer

Page 08

BH04

7. How much do you agree with the following statements about the ePA?

For each opinion, please indicate how strongly you agree with it.

	at all not yes	rather not	neutral	rather	fully and compl etely	No answer
I think that access to my ePA must be protected against misuse.	○	○	○	○	○	○
I think that security is more important than ease of use.	○	○	○	○	○	○
I think that registration should be quick and uncomplicated.	○	○	○	○	○	○
I think that creating my ePA should take less than 20 minutes.	○	○	○	○	○	○
I think that it should be possible to set up the ePA from home without visiting a branch of my health insurance company.	○	○	○	○	○	○
I think that training should be offered on the private use of the ePA	○	○	○	○	○	○
I think that my health insurance company should create my ePA at my request	○	○	○	○	○	○

Page 09

BH06

8. How much do you agree with the following statements?

Please indicate how strongly you agree with each statement.

	at all not yes	rather not	neutral	rather	fully and completely	No answer
In general, I am concerned about the privacy and security of my personal data in everyday life.	○	○	○	○	○	○
I am concerned about the privacy and security of my personal data when I use a mobile app.	○	○	○	○	○	○
I do not want my personal identifiers (such as name, telephone number, e-mail address, etc.) to be stored in save mobile apps.	○	○	○	○	○	○

Page 10

BH07

9. What security measures give you the confidence that an ePA protects your patient data?

Note: The term "mobile device" is used in the answer options. This refers to your cell phone, smartphone, tablet, laptop or similar device with which you can access the ePA.

☐ Encryption of the data stored on my mobile device and the data transmitted to a server ☐ User authentication (using password, PIN, facial recognition, etc.)

☐ Possibility of remote deletion of my personal data in the event of a lost mobile device ☐ Access control

☐ Easy-to-understand privacy policies that make it clear that my personal data is well protected ☐ Data transfer via a secure channel

☐ Easily adjustable security settings for different types of data

☐ Logging of all data access activities by medical staff so that access can be checked retrospectively

☐ Regularly update the password Other measures

☐ ____________

☐ I cannot assess these technical measures No answer

☐

EB01

Expectations and needs

The following section deals with expectations and needs that you associate with the electronic patient record.

10. How much do you agree with the following statements?

EB02

For each statement, please indicate how strongly you agree with it.

	at all not yes	rather not	neutral	rather	fully and compl etely	No answer
I expect the ePA to improve the quality of life of people with chronic illnesses.	○	○	○	○	○	○
I expect the ePA to have a positive impact on my quality of life.	○	○	○	○	○	○
I expect the ePA to increase my safety as a patient.	○	○	○	○	○	○
I expect the ePA to ensure my safety, especially in the event of an accident or medical emergency increased.	○	○	○	○	○	○
I expect my data to be adequately protected in the ePA.	○	○	○	○	○	○
I expect the ePA to be easy for me to use.	○	○	○	○	○	○
I expect the ePA to be easily accessible	fo	○	○	○	○	○

r me .○

EB03

11. Which of the following ePA functions do you consider useful?

Please rate how useful you think the functions listed are. Where 1 is not useful and 5 is very useful.

	not useful 1				useful 5	No answer
Making appointments with the family doctor and/or specialist Automatic	○	○	○	○	○	○
reminders of scheduled medical appointments Medication reminders	○	○	○	○	○	○
Reminders for preventive or early detection appointments (e.g. colonoscopy for cancer screening)	○	○	○	○	○	○
Transfer your own data from smartwatches/apps to the ePA, regardless of the device used	○	○	○	○	○	○
Easier exchange of my patient data between medical facilities (e.g. between my GP and my hospital)	○	○	○	○	○	○
Obtaining advice from medical experts (e.g. via a chat function)	○	○	○	○	○	○
Imparting knowledge about clinical pictures and health in general Imparting	○	○	○	○	○	○
knowledge about my specific clinical picture	○	○	○	○	○	○
Explanation of medical terms in patient-friendly language	○	○	○	○	○	○
Overview of my treatment and therapy history (e.g. completed medical appointments, medical findings)	○	○	○	○	○	○
Symptom diary	○	○	○	○	○	○
Alerting relatives and/or my doctor in the event of abnormal vital signs	○	○	○	○	○	○
Highlighted warning in the event of abnormal vital signs documented in the ePA	○	○	○	○	○	○
Collection of medical documents (e.g. X-rays, maternity record, vaccination record)	○	○	○	○	○	○
Collection of legal documents (e.g. organ donor card, patient decree, power of attorney for care)	○	○	○	○	○	○
Display of exercise videos (e.g. for back pain) Emergency	○	○	○	○	○	○
data (e.g. regular medication, allergies) Emergency contacts	○	○	○	○	○	○
(e.g. relatives with contact details)	○	○	○	○	○	○

EB04

12. What other function(s) would you consider useful?

☐ No answer

Page 13

EB05

13. How much do you agree with the following statements?

Please indicate how strongly you agree with each statement.

	at all not yes	rather not	neutral	rather	fully and compl etely	No answer
The ePA has a positive impact on my professional life.	◯	◯	◯	◯	◯	◯
The ePA places new complex demands on me.	◯	◯	◯	◯	◯	◯
The ePA makes it easier to treat patients.	◯	◯	◯	◯	◯	◯
The use of the ePA increases patient safety.	◯	◯	◯	◯	◯	◯
The ePA helps to save time on routine tasks (e.g. making appointments with patients).	◯	◯	◯	◯	◯	◯
Patient data is adequately otected in the ePA .◯	pr	◯	◯	◯	◯	◯
Patients find it easy to use their ePA.	◯	◯	◯	◯	◯	◯
The ePA simplifies communication with other service providers.	◯	◯	◯	◯	◯	◯
**If applicable:** As a medical practice, we are responsible for the creation and maintenance of the electronic health record. remunerated.	◯	◯	◯	◯	◯	◯

Page 14

EV01

Acquiring and teaching skills

The following section deals with whether and how you were informed about the electronic patient record and whether you would have liked more information.

EV02

14. Have you been informed about the ePA introduced in 2021?

○ Yes ○ No

3 active filter(s)

Filter EV02/F1
If one of the following answer option(s) was selected: **1**
Then show question/text **EV03** later in the questionnaire (otherwise hide)

Filter EV02/F2
If one of the following answer option(s) was selected: **1**
Then show question/text **EV04** later in the questionnaire (otherwise hide)

Filter EV02/F3
If one of the following answer option(s) was selected: **1**
Then show question/text **EV05** later in the questionnaire (otherwise hide)

Page 15

EV03

15. Who informed you about the ePA?

Multiple answers possible

- ☐ Doctor
- ☐ Health insurance
- ☐ Federal Ministry of Health☐ Employer
- ☐ Family members or acquaintances
- ☐ As part of my training or professional qualification☐ Media
- ☐ Other ______

☐ No answer

Page 16

EV04

16. In what form were you informed?

Multiple answers possible

- [ ] Information brochure(s) in printed form
- [ ] Electronically in writing, i.e. by e-mail
- [ ] Electronically digital, i.e. via overlay in an app
- [ ] Online information campaign, i.e. via banner insertion
- [ ] Radio or TV
- [x] **Social media**
  - [ ] Twitter
  - [ ] Instagram
  - [ ] Facebook
  - [ ] YouTube
  - [ ] Other: ________
- [ ] Other: ________

- [ ] No answer

Page 17

EV05

17. What specific ePA information material was available to you?

Multiple answers possible

- [ ] Brochure (paper)
- [ ] Flyer (paper)
- [ ] Information letter
- [ ] Technical article (electronic / paper)
- [ ] Video(s)
- [ ] Social media posts
- [ ] Other ________

- [ ] No answer

EV06

18. How much do you agree with the following statements?

For each statement, please indicate how much you agree with it.

	at all not yes	rather not	neutral	rather	fully and compl etely	No answer
As a **patient**, I feel **involved** in the introduction of the ePA	○	○	○	○	○	○
As a **citizen**, I feel **involved** in the introduction of the ePA	○	○	○	○	○	○
As a **specialist**, I feel **involved** in the introduction of the ePA	○	○	○	○	○	○
As a **patient**, I feel **informed** about the introduction of the ePA	○	○	○	○	○	○
As a **citizen**, I feel **informed** about the introduction of the ePA	○	○	○	○	○	○
As a **specialist**, I feel **informed** about the introduction of the ePA	○	○	○	○	○	○

EV07

19. How would you like to be informed so that you feel sufficiently well informed about the ePA?

Multiple answers possible

By talking to ☐ a doctor ☐
a health insurance company
☐ my employer
☐ Persons within the scope of my education or professional qualification ☐
Family members, e.g. grandparents/parents, siblings, children, etc.
☐ Acquaintances or friends
☐ Other:

In writing, in
☐ in printed form, e.g. by information letter or flyer ☐ in electronic form, i.e. by e-mail
☐ Other:

Digital, per
☐ My health insurance company's app ☐ Social media campaign
☐ Multimedia offer, e.g. video ☐
Other:

Through media offerings, in
☐ Radio
☐ TV
☐ Podcast
☐ Newspapers / magazines
☐ Other:

☐ No answer

Page 20

EV09

20. Who have you already spoken to about the possibilities of using the ePA?

Multiple answers possible

- [ ] Doctor
- [ ] Health insurance company
- [ ] Family members, e.g. grandparents/parents, siblings, children, etc.
- [ ] Acquaintances or friends
- [ ] Work colleagues
- [ ] Other

- [ ] None of the above
- [ ] No answer

Page 21

EV10

21. Which of the following people or institutions have informed you about using the ePA?

Multiple answers possible

- [ ] Doctor
- [ ] Health insurance company
- [ ] Family members, e.g. grandparents/parents, siblings, children, etc.
- [ ] Acquaintances or friends
- [ ] Work colleagues
- [ ] Other

- [ ] None of the above
- [ ] No answer

Page 22

Demographic data
Personal data is requested in the following section. These questions are formulated in such a way that you cannot be identified on the basis of your details.

22. How old are you? SD02

- ◯ 18-29
- ◯ 30-39
- ◯ 40-49
- ◯ 50-59
- ◯ 60-69
- ◯ 70-79
- ◯ 80 years and older

◯ No answer

Page 23

23. What is your highest educational qualification? SD03

◯ No school leaving certificate
◯ Elementary/main school certificate◯ Realschule (Mittlere Reife)
◯ Completion of an apprenticeship or vocational school◯ Abitur
◯ Technical/university degree
◯ Degree from a university of applied sciences or a university of cooperative education◯ Doctorate

---

◯ No answer

Page 24

SD04

24. What is your gender?

◯ Female◯
Male
◯ Non-binary / genderqueer◯
No gender
◯ Own information ____

◯ Not specified

Page 25

SD05

25. What is your employment status?

◯ Employed
◯ In training
◯ Retired / retiring◯
Not employed
◯ Self-employed / freelance◯
Civil servant
◯ Miscellaneous

◯ No answer

Page 26

SD06

26. How many known pre-existing conditions do you have?

◯ None◯
1
◯ 2
◯ 3
◯ 4 or more

◯ No answer

SD07

27. How do you have health insurance?

◯ Statutory◯
Private

◯ No answer

Page 27

SD08

28. Do you have any comments that you would like to share with us?

Are you interested in receiving the results of the survey by e-mail? Then write to ag.chi@gmds.de

Last page

Thank you for your participation!

We would like to thank you very much for your help.

Your answers have been saved, you can now close the browser window.

GMDS AG Consumer Health Informatics (CHI) - 2021
