# Supplementary material for: Consumer perspectives on the national electronic health record and barriers to its adoption in Germany: does health policy require a change in communication?
Source: BMC Health Serv Res. 2025 Jan 7;25:33. doi: 10.1186/s12913-024-12175-6 (PMC11706193; doi:10.1186/s12913-024-12175-6)
Supplement: Supplementary file 2 — Additional file 2. Mapping of German Education Levels to International Standard Classification of Education (ISCED). [file 12913_2024_12175_MOESM2_ESM.docx]

## Additional file 2 - Mapping of German Education Levels to International Standard Classification of Education (ISCED)

**Source:** Federal Statistical Office, “Bildungsfinanzbericht 2021”, Anhang A2 (Appendix A2)

| **ISCED level Category** | **Sub-category** | **German Education programmes** |
| --- | --- | --- |
| **ISCED 0 Early childhood education** | | |
| ISCED 01 Early childhood educational development for children younger than three years | 010 | Crèches |
|  | 010 | Day care centres for children under 3 years |
| ISCED 02 Pre-primary education for children from the age of three years to the start of primary education | 020 | Kindergartens |
|  | 020 | Pre-school classes |
|  | 020 | School kindergartens |
|  | 020 | Day care centres for children in kindergarten age |
| ISCED 1 Primary education | | |
| ISCED 10 general education | 100 | Primary schools |
|  | 100 | Comprehensive schools (grades 1-4) |
|  | 100 | Waldorf schools (grades 1-4) |
|  | 100 | Special needs schools (grades 1-4) |
| ISCED 2 Lower secondary education | | |
| ISCED 24 general education | 241 | Orientation stages (grades 5/6) |
|  | 244 | Secondary general schools |
|  | 244 | Intermediate schools |
|  | 244 | Special needs schools (grades 5-10) |
|  | 244 | Schools with different courses of education |
|  | 244 | Grammar schools (grades 5-9/10)1 |
|  | 244 | Comprehensive schools (grades 5-9/10)1 |
|  | 244 | Waldorf schools (grades 5-10) |
|  | 244 | Evening secondary general schools |
|  | 244 | Evening intermediate schools |
|  | 244 | Catch up on lower secondary school degrees |
|  | 244 | Fulfilment of compulsory education at vocational schools |
|  | 244 | Vocational schools that lead to intermediate degrees |
| ISCED 25 vocational education | 254 | Pre-vocational training year (and other vocational preparation programmes, e.g. at part-time or fulltime vocational schools) |
| **ISCED 3 Upper secondary education** | | |
| ISCED 34 general education | 344 | Grammar schools (grades 10-12/11-13)1 |
|  | 344 | Comprehensive schools (grades 10-12/11-13)1 |
|  | 344 | Waldorf Schools (grades 11-13) |
|  | 344 | Special needs schools (grades 11-13) |
|  | 344 | Specialised upper secondary schools – two-years (without previous vocational education) |
|  | 344 | Specialised grammar schools |
|  | 344 | Full-time vocational schools that lead to higher education entry qualification |
| ISCED 35 vocational education | 351 | Basic vocational training year (and other basic vocational programmes that are taken into account to the first year of apprenticeship) |
|  | 353 | One-year programmes at training institutions/schools for health care and social professions |
|  | 353 | Completion of the preparatory service for the middle grade in public administration |
|  | 354 | Part-time vocational schools (dual system) - initial training |
|  | 354 | Full-time vocational schools that lead to a vocational certificate (excluding health care and social professions and educator training) |
| ISCED 4 Post-secondary non-tertiary education | | |
| ISCED 44 general education | 444 | Evening grammar schools, adult education colleges |
|  | 444 | Specialised upper secondary schools – one-year (after previous vocational education) |
|  | 444 | Two-year full-time vocational schools |
| ISCED 45 vocational education | 453 | Two- and three-year programmes at training institutions/schools for health care and social professions |
|  | 454 | Part-time vocational schools (dual system) (second education after obtaining a higher education entrance qualification)2 |
|  | 454 | Full-time vocational schools that lead to a vocational certificate (second education after obtaining a higher education entrance qualification)2 |
|  | 454 | Vocational programmes that lead to a higher education entry qualification as well as to a vocational certificate (simultaneously or successively)2 |
|  | 454 | Part-time vocational schools (dual system) (second education, vocational) |
|  | 454 | Part-time vocational schools (dual system) – retrainees |
| ISCED 5 Short-cycle tertiary education | | |
| ISCED 54 general education | - |  |
| ISCED 55 vocational education | 554 | Master craftsman training (very short preparation courses only that last less than 880 hours)3 |
| ISCED 6 Bachelor’s or equivalent level | | |
| ISCED 64 academic | 645 | Bachelor's programme at  Universities (scientific institutions of higher education including colleges of education, colleges of theology, and colleges of art and music)  Universities of applied sciences (including schools of engineering)  Cooperative state universities of Baden-Württemberg and Thuringia  Colleges of public administration  Vocational academies |
|  | 647 | Diplom degree programme at universities of applied sciences |
|  | 647 | Diplom degree programme at colleges of public administration |
|  | 647 | Diplom degree programme at vocational academies |
|  | 647 | Second Diplom degree programme |
|  | 647 | Second Diplom degree programme at universities of applied sciences |
| ISCED 65 professional | 655 | Trade and technical schools, e.g. business economist, business administrator (excluding health care and social professions and educator training) |
|  | 655 | Master craftsman training (preparation courses lasting 880 hours or more) |
|  | 655 | Training institutions/schools for educators |
|  | 655 | Specialised academies (Bavaria) |
| ISCED 7 Master’s or equivalent level | | |
| ISCED 74 academic | 746 | Diplom degree programme at university (including teacher training, state examination, Magister programme, artistic and comparable programmes) |
|  | 747 | Master's programme at  Universities (scientific institutions of higher education including colleges of education, colleges of theology, and colleges of art and music)  Universities of applied sciences (including schools of engineering)  Baden-Württemberg and Thuringia Cooperative State Universities  Colleges of public administration |
|  | 748 | Second master's programme |
|  | 748 | Second Diplom degree programme at university |
| ISCED 75 professional | - |  |
| ISCED 8 Doctoral or equivalent level | | |
| ISCED 84 academic | 844 | Doctoral studies |
| ISCED 9 No other classification | | |
| ISCED 99 No other classification | 999 | Pupils at special needs schools that cannot be assigned to an education area |
|  | 999 | No assignment to a school type possible4 |

**1)** At grammar schools and integrated comprehensive schools leading to a school-leaving certificate after grade 12, upper secondary level begins at grade 10 instead of 11.

**2)** Fully qualifying vocational programmes after obtaining a higher education entrance qualification or fully qualifying vocational programmes that additionally lead to a higher education entrance qualification get allocated to ISCED 454 pursuant to Eurostat definition.

**3)** The allocation is based on the subject field of the preparation courses.

**4)** In Hesse: foreign pupils, that gain basic German language skills in intensive classes.

Notes on sub-categories

^241^ Insufficient for level completion or partial level completion, without direct access to upper secondary education.

^244,254 351^ Level completion, with direct access to upper secondary education.
Insufficient for level completion or partial level completion, without direct access to tertiary education.

^353^ Level completion, without direct access to first tertiary programmes (but may give direct access to postsecondary non-tertiary education).

^344,354^ Level completion, with direct access to first tertiary programmes (may also give direct access to postsecondary non-tertiary education).

^453^ Level completion, without direct access to first tertiary programmes.

^444,454^ Level completion, with direct access to first tertiary programmes.
